# Isolation of Biofilm-Forming Staphylococci from the Bulk-Tank Milk of Small Ruminant Farms in Greece

**DOI:** 10.3390/foods12152836

**Published:** 2023-07-26

**Authors:** Daphne T. Lianou, Charalambia K. Michael, Nikolaos Solomakos, Natalia G. C. Vasileiou, Efthymia Petinaki, Vasia S. Mavrogianni, Athina Tzora, Chrysoula Voidarou, George C. Fthenakis

**Affiliations:** 1 Veterinary Faculty, University of Thessaly, 43100 Karditsa, Greece; 2 Faculty of Animal Science, University of Thessaly, 41110 Larissa, Greece; 3 University Hospital of Larissa, University of Thessaly, 41110 Larissa, Greece; 4 Faculty of Agriculture, University of Ioannina, 47100 Arta, Greece

**Keywords:** biofilm, goat, mastitis, milk, sheep, slime, somatic cell counts, *Staphylococcus*

## Abstract

The objectives of this study were (i) to describe staphylococcal isolates recovered from bulk-tank raw milk collected from sheep and goat farms during a countrywide study performed in Greece, (ii) to study management factors potentially associated with their presence in bulk-tank milk and (iii) to provide evidence regarding their association with the quality of the milk. In total, 312 staphylococcal isolates, recovered from samples of bulk-tank raw milk from 444 small ruminant farms in Greece, were evaluated in this work. The in vitro formation of biofilm by the isolates was tested by combining the findings of (a) culture appearance on Congo Red agar plates and (b) results of a microplate adhesion test. The most frequently identified species was *Staphylococcus aureus* (75 isolates); other frequently recovered species were *S. simulans* (44 isolates), *S. equorum* (34 isolates) and *S. haemolyticus* (26 isolates); in total, 23 species were identified. In total, 224 (71.8%) isolates were biofilm-forming and were recovered from the bulk-tank milk samples of 148 sheep flocks (45.5%) and 55 goat herds (46.2%). There was evidence of seasonality in the isolation of staphylococci: during spring, mostly biofilm-forming isolates were recovered, whilst during summer, mostly non-biofilm-forming isolates were recovered. Among farms applying machine-milking, the proportion of farms from which biofilm-forming isolates were recovered was higher where water with temperature < 50 °C or ≥90 °C was used to clean the milking parlour. In the multivariable analyses, for farms applying machine-milking, the temperature of the water emerged as the only significant variable (*p* = 0.024), whilst in farms applying hand-milking, the only tendency that emerged was for the frequency of collection of milk from the farm tank (*p* = 0.08). In sheep flocks, recovery of biofilm-forming staphylococci from the bulk-tank milk was associated with higher somatic cell counts and higher total bacterial counts in the milk. The study identified abiotic factors related to the presence and isolation of these bacteria, specifically the temperature of water used for the cleaning of the milking parlour (in farms where machine-milking is applied) and the frequency of milk collection from the farm tank. These factors apply after the production of milk, and they could thus be regulated appropriately in order to reduce bacterial load and improve the quality of milk delivered to dairy plants. In sheep farms, an association was also seen between recovery of biofilm-forming staphylococci and high somatic cell counts in milk.

## 1. Introduction

Biofilm formation is a process through which bacteria attach onto a surface and produce extracellular polymers, thus making this attachment easier, as well as matrix formation [1]. The result is a change in the phenotypic characteristics of the bacteria. Biofilm formation is an important mechanism in the pathogenesis of diseases, as infections associated with biofilm-forming bacteria do not respond well to antimicrobial treatments [2].

Staphylococci are the predominant causal agents of mastitis in small ruminants [3]. Biofilm formation by staphylococci is an important virulence factor of these organisms [4]. First, it allows dissemination onto the teatcups and the liners in the parlour, that way increasing the risk of infecting animals during milking; in a recent study of staphylococcal populations on the teatcups of milking parlours in sheep and goat farms, over 80% of the isolates recovered have been found to be biofilm-forming [5]. Further, within the mammary gland, biofilm formation facilitates expansion of the multiplying bacteria after the initial infection, which contributes to extending lesions within the mammary parenchyma. Finally, the ability to form biofilm is associated with reduced susceptibility to antibiotics, which thus makes these bacteria difficult responders to antibiotic treatment [6,7].

The first detailed study of biofilm formation by mastitis-related staphylococcal strains is the one performed by Vautor et al. [8]. Moreover, a variety of relevant works have been published. A recent topic search in the Web of Science platform by using the term string [[sheep OR goat*] AND [staphylococcus] AND [biofilm OR slime] AND [milk OR mastitis]] and the subsequent assessment of the references individually indicated a total of 68 relevant articles. Most of these studies have presented the characteristics of staphylococcal strains from cases of mastitis and confirmed the increased frequency of isolation of these organisms from cases of the infection. Vasileiou et al. [9] have described ‘mastitis caused by biofilm-forming staphylococci’ as a new disease entity, i.e., based on the presence of this virulence characteristic rather than the species identity of the strains. Perez et al. [4] have developed of a vaccine against ovine and caprine mastitis caused by biofilm-forming staphylococci, which further indicates the importance of these bacteria as mammary pathogens. More recent studies are dealing with the characterisation of the genes regulating biofilm-formation by staphylococcal isolates recovered from milk [10,11,12].

There is nevertheless a scarcity of data regarding biofilm characterisation of staphylococci recovered from the bulk-tank raw milk from sheep and goats. Hence, the objectives of this study were (i) to describe staphylococcal isolates recovered from bulk-tank raw milk collected from sheep and goat farms during a countrywide study performed in Greece, (ii) to evaluate factors potentially associated with the presence of the staphylococci in bulk-tank milk and (iii) to provide evidence regarding potential association of these bacteria with the quality of the milk. This work presents the identification of biofilm-forming staphylococci in the bulk-tank milk of sheep and goat farms and also evaluates some factors that may be associated with the presence of these bacteria. The findings are important for the small ruminant dairy industry, particularly in the para-Mediterranean countries, where the largest proportions of ovine and caprine milk in Europe are produced.

## 2. Materials and Methods

### 2.1. Visits to Sheep and Goat Farms

**Figure 1:**
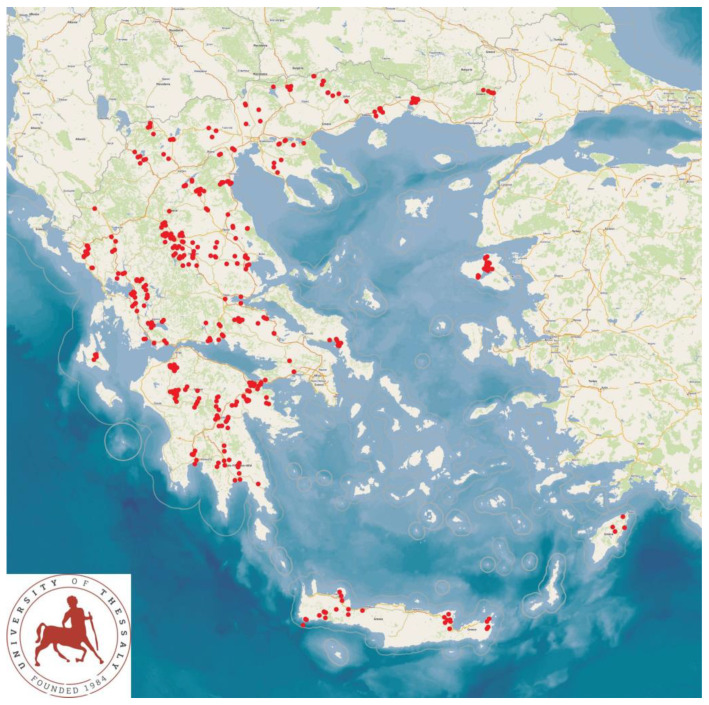
Locations (red dots) of 444 small ruminant farms around Greece, in the 13 administrative regions of the country, which were visited during a countrywide investigation for bulk-tank milk sampling for recovery of staphylococcal isolates.

A cross-sectional study was performed during a countrywide investigation in sheep flocks and goat herds in Greece. The study was performed from April 2019 to July 2020. During the study, 444 farms (325 with sheep and 119 with goats) were visited. The farms were located in all 13 administrative regions of the country, in order to provide a wide territorial coverage (Figure 1). Investigators carried out on-site visits to all the farms included in the study.

During the visits, information was obtained from the shepherds and the goatherds regarding their farms and the management practices applied in these farms (Appendix A), by means of an interview using a structured questionnaire [13,14].

Moreover, milk samples were collected from the bulk-tank of each farm. Four 20 mL samples were collected from each bulk-tank. Standard aseptic conditions were obtained for sampling, e.g., use of gloves by the investigators, use of plastic disposable pipettes for withdrawal of milk, thorough mixing of the milk within the bulk tank, use of plastic disposable containers, etc.

Samples were stored at 0.0 to 4.0 °C using ice packs in portable refrigerators. Somatic cell counting and milk composition measurement were performed on each of the samples within 4 h of sample collection. Transportation of samples to the laboratory was carried out by the investigators and by car; samples collected from farms in the islands of the country were transported as accompanying luggage by boat or by airplane.

### 2.2. Laboratory Examinations

Initially, measurement of milk composition (Lactoscan Farm Eco; Milkotronic Ltd., Nova Zagora, Bulgaria) and somatic cell counting (Lactoscan SCC; Milkotronic Ltd., Nova Zagora, Bulgaria) were performed. These were carried out in duplicate in each of two milk samples collected from the bulk-tank.

Bacteriological examinations followed within 24 h. Total bacterial counts in the milk samples were performed also in duplicate in the other two samples collected from the bulk-tank. The procedures detailed by Laird et al. [15] were followed; plates were incubated at 37 °C for 48 h and were thereafter counted within 2 h. Milk samples (10 μL) were also cultured in duplicate on staphylococcus selective medium (Mannitol salt agar; BioPrepare Microbiology, Athens, Greece) (i.e., from each of two samples collected, two technical samples were processed for bacteriological examination); all plates were incubated aerobically at 37 °C for 48 h; if there was no growth, plates were re-incubated for another 24 h. After completion of sample aliquot withdrawal for microbiological examination, the temperature of the respective samples was measured and in no case was found to exceed 3.8 °C. Bacterial isolation and initial identification were performed using standard methods [16,17]. Detection of at least three confirmed staphylococcal colonies on at least one agar plate of the four inoculated with each bulk-tank milk sample from each flock, was considered to indicate presence of the organism.

After initial isolation, identification of staphylococci at species level was carried out by means of Matrix-Assisted Laser Desorption/Ionization Time-of-Flight Mass Spectrometry (VITEK MS; BioMerieux, Marcy-l’-Étoile, France). Subsequently, the in vitro biofilm formation by the isolates was tested by combining the findings of (a) culture appearance on Congo Red agar plates and (b) results of microplate adhesion test, as detailed by Vasileiou et al. [9]. In order to assess culture appearance of staphylococcal isolates, these were cultured on Congo Red agar plates (BioPrepare Microbiology, Athens, Greece), which were incubated aerobically at 37 °C for 24 h [18]. For the assessment of biofilm formation by means of the microplate method, the technique presented by Fabres-Klein et al. [19] was followed, based on the principles set by Vasudevan et al. [20]; the method involved the measurement of the absorbance rate after coating microplate wells with bacterial culture incubated with tryptic soy broth [19]. The results of the two methods were combined [9], and the isolates were characterised as biofilm-forming or non-biofilm-forming.

### 2.3. Data Management and Analysis

Data were entered into Microsoft Excel and analysed using SPSS v. 21 (IBM Analytics, Armonk, NY, USA). Basic descriptive analysis was performed. Exact binomial confidence intervals (CIs) were obtained. For the statistical analysis, somatic cell counts were transformed as previously detailed by Wiggans and Shook [21] and Franzoi et al. [22]: somatic cell scores = log_2_(somatic cell counts/100) + 3; total bacterial counts were transformed to log_10_. The transformed data were used in the analyses; back-transformation of the results obtained was carried out for the presentation of the results.

The outcome ‘isolation of biofilm-forming staphylococci’ was considered. Eleven parameters related to farm management (Appendix A), as well as the season when the visit to each farm and the sampling were performed, were evaluated for potential association with this outcome. Exact binomial CIs were obtained. For each of these parameters, categories were created according to the replies of the farmers. Initially, the importance of predictors was evaluated by using cross-tabulation with Pearson’s chi-square test (for categorical variables with *n* > 5) or Fisher exact test (for categorical variables with *n* ≤ 5) or Mann–Whitney test (for continuous variables with no normal distribution) and with simple logistic regression without random effects.

Then, a multivariable model was developed for the above outcome and parameters, found with *p* < 0.2 in the preceding univariable analyses, were offered to this model. Progressively, variables were removed from the model by using backwards elimination. The likelihood ratio test was performed to assess the *p*-value of each parameter; among those found with *p* > 0.2, the one with the largest *p* was removed from the model. The procedure was repeated, until no variable with *p* > 0.2 could be removed from the model. Two different models were developed and separate analyses were carried out, one for farms applying machine-milking and one for farms applying hand-milking, as one of the variables found with *p* < 0.05 in the univariable analysis was related to the type of milking and thus could not be applied uniformly in all farms in the study. The variables included in the final multivariable models constructed, are detailed in Table S1.

Potential associations between isolation of biofilm-forming staphylococci from bulk-tank milk and milk quality-related parameters were assessed by using one-way analysis of variance to compare between farms where biofilm-forming staphylococci were isolated, farms where non-biofilm-forming staphylococci were isolated and farms where no staphylococci were isolated. Then, the Tukey procedure was employed to identify differences between pairs of groups.

Statistical significance was defined as *p* < 0.05.

## 3. Results

The data collected during the visits are summarised in Table S2, classified according to animal species (sheep or goats) in the farms.

### 3.1. Staphylococcal Recovery and Identity

A total of 312 staphylococcal isolates were recovered during the study. Staphylococci were recovered from the bulk-tank milk samples of 206 sheep flocks (63.4%, 95% CI: 58.0–68.4%) and of 75 goat herds (63.0%, 95% CI: 54.1–71.2%). There was no significant difference in the frequency of isolation of staphylococci between sheep and goat farms (*p* = 0.94).

The median number of staphylococcal isolates recovered from milk samples was 1 (interquartile range: 1). There was no significant difference in the median number of isolates recovered from sheep or goat farms: 1 (interquartile range: 1) for both (*p* = 0.72).

In total, 23 species were identified in the samples. The most frequently identified species was *Staphylococcus aureus* with 75 isolates in total (54 from sheep and 21 from goat (*p* = 0.17) farms; 51 from machine-milked and 24 from hand-milked (*p* = 0.38) farms). Other frequently recovered species were *S. simulans* (*n* = 44 isolates in total), *S. equorum* (*n* = 34 isolates) and *S. haemolyticus* (*n* = 26 isolates). There were no differences in the frequency of the various staphylococcal isolates recovered from sheep or goat farms and from farms with machine- or hand-milking (*p* > 0.06), bar for the frequency of isolation of *S. pettenkoferi* between farms with sheep (from 0.0% of flocks) or goats (from 3.8% of herds) (*p* = 0.004). Details of the identities of the isolates are in Table 1.

### 3.2. Biofilm Formation by Staphylococcal Isolates

**Figure 2:**
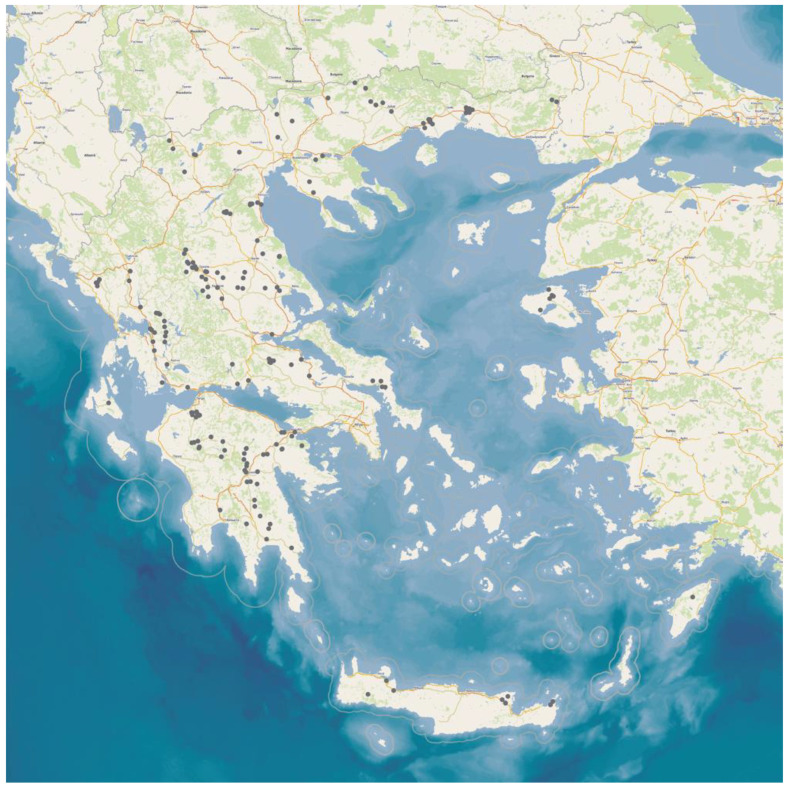
Location (black dots) of small ruminant farms (*n* = 203) around Greece, from the bulk-tank milk of which biofilm-forming staphylococcal isolates were recovered during a countrywide investigation.

In total, 224 (71.8%) isolates were found to be biofilm-forming. The median number of biofilm-forming staphylococcal isolates recovered from milk samples was 0 (interquartile range: 1). These isolates were recovered from the bulk-tank milk samples of 203 farms (Figure 2), specifically from 148 sheep flocks (45.5% of all flocks, 71.8% of flocks from which staphylococci were isolated from the bulk-tank milk) and 55 goat herds (46.2% of all herds, 73.3% of herds from which staphylococci were isolated from the bulk-tank milk). There was no significant difference in the proportion of sheep flocks and goat herds from which biofilm-forming staphylococci were isolated (*p* = 0.90).

No differences were seen in biofilm formation by isolates in accordance with animal species or milking-mode: 166/232 (71.6%) from sheep and 58/80 (72.5%) from goat farms (*p* = 0.87) and 164/227 (72.2%) from farms with machine- and 60/85 (70.6%) from farms with hand-milking (*p* = 0.77). There was no significant difference in the median number of biofilm-forming isolates recovered from sheep or goat farms and in the median number of biofilm-forming isolates recovered from farms applying machine- or hand-milking (0 (interquartile range: 1) for all (*p* > 0.84 for all comparisons).

There was some difference in the proportion of isolates that were biofilm-forming in accordance with the species (*p* = 0.042). All *S. auricularis*, *S. carnosus*, *S. pasteuri*, *S. pettenkoferi*, *S. saprophyticus* and *S. sciuri* isolates studied were biofilm-forming; the lowest proportion of biofilm-forming isolates was recorded among *S. haemolyticus* (46.2%), *S. cohnii* subsp. *cohnii* (40.0%) and *S. lugdunensis* (38.5%) (Table 2).

### 3.3. Variables Associated with Isolation of Biofilm-Forming Staphylococci

**Figure 3:**
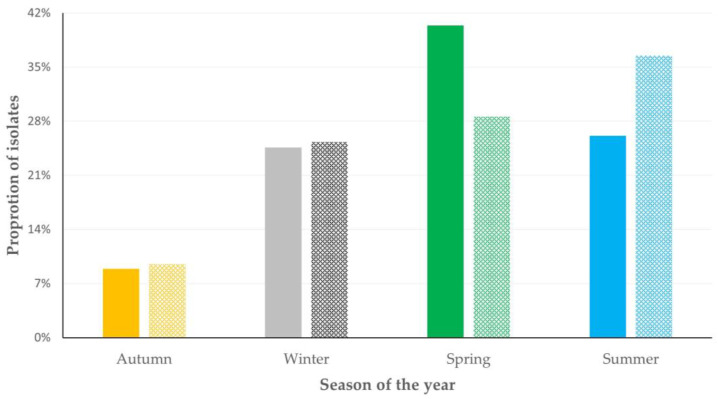
Proportion of farms from which biofilm-forming staphylococcal isolates were (solid bars) or were not (pattern bars) recovered, in accord with the season of the year when the visit was made: during spring, isolation of biofilm-forming isolates from 54.3% of farms visited during that season, during summer, isolation of non-biofilm forming isolates from 62.4% of farms visited during that season.

There was evidence of seasonality in the isolation of staphylococci. During the spring, mostly biofilm-forming isolates were recovered: from 54.3% of farms visited during that season and from 40.4% of farms from which biofilm-forming isolates were recovered (*p* = 0.009 when compared with the other seasons). During the summer, mostly non-biofilm forming isolates were recovered: from 62.4% of farms visited during that season and from 36.5% of farms from which non-biofilm-forming isolates were recovered (*p* = 0.019 when compared with the other seasons) (Figure 3, Table S3).

**Figure 4:**
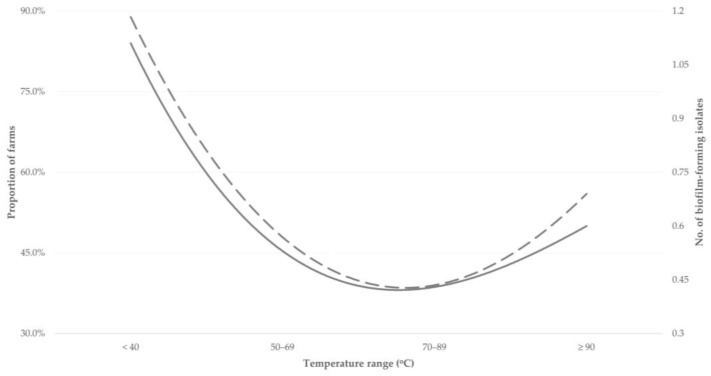
Proportion of farms applying machine-milking, from which biofilm-forming staphylococcal isolates were recovered from bulk-tank milk (dashed line) and average number of isolates from these farms (solid line), in accord with the temperature of the water used to clean the milking parlour (proportion of farms from which biofilm-forming staphylococci were isolated according to temperature ranges <49 °C: 88.9%, 50–69 °C: 47.9%, 70–89 °C: 39.0%, ≥90 °C: 56.0%; median number of biofilm-forming staphylococci isolated per farm according to temperature ranges <49 °C: 1, 50–69 °C: 0, 70–89 °C: 0, ≥90 °C: 1).

Among farms applying machine-milking, the proportion of those in which biofilm-forming isolates were recovered was significantly higher in those where water with temperature < 50 °C was used for the cleaning of the milking parlour: 88.9% of these farms (*p* = 0.0002); in these farms, the median number of biofilm-forming isolates recovered from the bulk-tank milk was also significantly higher: 1 (0) (*p* < 0.0001) (Figure 4, Table S4). The proportion of farms from which biofilm-forming isolates were recovered, was significantly lower where water with a temperature 50 °C to 89 °C was used: 39.0% of these farms (*p* = 0.005); in these farms, the median number of biofilm-forming isolates recovered from the bulk-tank milk was also significantly higher: 0 (1) (*p* = 0.005) (Figure 4, Table S4).

There was also a tendency for association between the isolation of biofilm-forming staphylococci and the frequency of milk collection from the farm tank; milk collections were carried out less frequently (on average, every 1.85 ± 0.04 days) in farms where biofilm-forming staphylococci were isolated than in farms where they were not (on average, every 1.74 ± 0.03 days) (*p* = 0.05). With regard to other management practices in the farms, no associations with recovery of biofilm-forming isolates were identified (*p* > 0.10 for all comparisons) (Table S5).

In the multivariable analyses, for farms applying machine-milking, the temperature of the water used to clean the milking parlour emerged as the only significant variable (*p* = 0.024) for the isolation of biofilm-forming staphylococci (Table 3). In contrast, in farms applying hand-milking, only a tendency emerged for the frequency of collection of milk from the farm tank (*p* = 0.08) (Table 3).

### 3.4. Association of Recovery of Biofilm Formation by Staphylococcal Isolates with Quality of Bulk-Tank Milk

**Figure 5:**
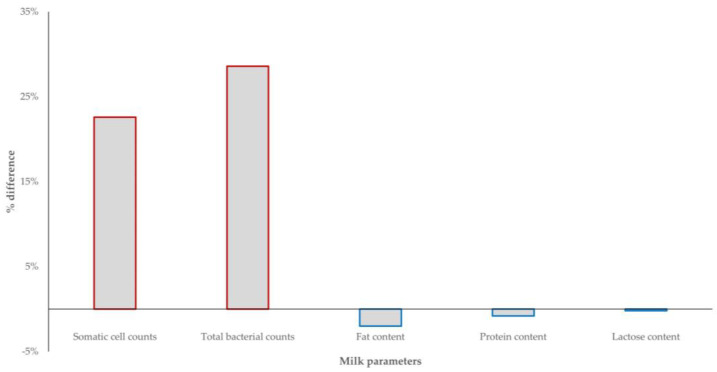
Differences (%) in overall results of quality-related parameters (somatic cell counts, total bacterial counts, fat content, protein content, lactose content) of the bulk-tank in samples from which biofilm-forming staphylococci were isolated in comparison to samples from which biofilm-forming staphylococci were not isolated (*p*-values: for somatic cell counts 0.0002, for total bacterial counts 0.028, for fat content 0.29, for protein content 0.56, for lactose content 0.83).

There was evidence that, in sheep flocks, recovery of biofilm-forming staphylococci from the bulk-tank milk was associated with higher somatic cell counts and higher total bacterial counts in the milk; however, there were no differences in the chemical composition of milk in accord with the recovery of biofilm-forming isolates. In goat herds, no significant differences were seen in any parameter (Figure 5, Table S6).

In sheep flocks, among samples from which biofilm-forming staphylococci were isolated, those from which *S. aureus* was recovered, had higher somatic cell counts than those from which non-*aureus* isolates were recovered: 0.684 × 10^6^ (0.556 × 10^6^–0.848 × 10^6^) versus 0.537 × 10^6^ (0.477 × 10^6^–0.608 × 10^6^) cells mL^−1^ (*p* = 0.047). In contrast, among samples from which non-biofilm-forming staphylococci were isolated, the respective difference was not significant: 0.617 × 10^6^ (0.427 × 10^6^–0.890 × 10^6^) versus 0.474 × 10^6^ (0.401 × 10^6^–0.560 × 10^6^) cells mL^−1^ (*p* = 0.17).

## 4. Discussion

Most of the staphylococcal isolates recovered from the bulk-tank milk samples (71.5% overall) were found to be biofilm-forming. This is in line with the findings of Vasileiou et al. [9], who reported that 69% of staphylococcal isolates causing mastitis in sheep were biofilm-forming. In this respect, the observed association between increased somatic cell counts and isolation of biofilm-forming staphylococci is likely the consequence of subclinical mastitis, caused by bacterial isolates in the animals of the farms; the infection leads to mammary inflammation and high somatic cell counts. The finding of higher somatic cell counts in milk from which *S. aureus* was recovered, in comparison to milk from which non-*aureus* isolates were obtained, is in line with the increased pathogenetic effects of the former bacteria, which elicit a more intense inflammatory response from the animals and thus lead to higher somatic cell counts. This suggests that most of the isolates were likely of animal origin.

Nevertheless, staphylococci recovered from the bulk-tank milk also include (i.e., additionally to isolates from the milk of animals) isolates of environmental (e.g., from the pipelines of the milking system) or human (e.g., from people working in the farm) origin. This becomes evident when considering the identity of the isolates, for example, the isolation of *S. carnosus* from cases of mastitis has not been reported [23], but this species (originally isolated from meat products [24]) was identified in the current set of isolates. Moreover, in previous papers, we have reported the isolation of fosfomycin-resistant isolates [25,26], which we postulated to be of human origin, given that fosfomycin is not licenced for veterinary use and actually is not used for animal therapeutics in Greece.

There is interest in identifying factors prevailing after milking the animals, which can influence the presence of bacteria in the raw milk before it is collected by dairy companies. In the dairy industry, a cause of bacterial cross-contamination is the formation of biofilm during milk storage and processing, as the organisms adhere onto stainless steel surfaces [27], including the milk tank. During milk processing and dairy product manufacturing, biofilm formation by bacteria may lead to potential problems. Biofilm-forming bacteria may survive during some heat-processing steps (e.g., high temperature pasteurisation) [28]. In dairy production plants, survival of biofilm-forming bacteria can lead to the contamination of equipment, which is mostly made of stainless steel [29], whilst the bacteria can be further transmitted to cheese and other dairy products [30]. It is also notable that biofilm formation by *S. aureus* can be enhanced through some of the processing methods employed in the dairy food industry, among which is the use of salt during cheese manufacturing [31]. The above can pose risks for consumers of dairy products and, moreover, they can affect the quality of cheese produced [32].

In farms where machine-milking is employed, the use of cleaning water for cleaning of the milking parlour, with temperatures within the range of 70 °C to 90 °C contributes to reducing the presence of biofilm-forming staphylococci in the bulk-tank milk, as well as the number of staphylococcal isolates therein. In view of this, water with such temperatures should be used for the cleaning procedure. This needs to be applied diligently, as it emerged to be the most important factor influencing the presence of staphylococci in raw milk. In farms where hand-milking is applied, the frequency of milk collection from the farm can be increased; if milk collection is delayed, staphylococci are allowed to attach on the surface of the containers, where milk is collected, and to form biofilm, which can protect them from adverse environmental conditions (e.g., disinfectants). In this respect, frequent emptying of the containers contributes to reducing the risk of bacterial colonisation and biofilm formation, as the tendency observed in the present study. The seasonality in the frequency of recovery of biofilm-forming isolates can be explained through the behaviour of bacteria under different environmental conditions: increased relative humidity can promote biofilm formation [33], which is aligned with high frequency of isolation during the spring and the lowest frequency during the dry Greek summer [34]. This seasonality may also be taken into account when regulating the temperature of the water used to clean the milking parlour.

## 5. Conclusions

Approximately 72% of staphylococcal isolates recovered from bulk-tank milk from small ruminant farms in Greece were biofilm-forming. Most frequent biofilm-forming species were *S. aureus*, *S. simulans*, *S. equorum* and *S. haemolyticus*. The study identified abiotic factors related to the presence and isolation of these bacteria, specifically the temperature of water used to clean the milking parlour (in farms where machine-milking is applied) and the frequency of milk collection from relevant containers. These factors apply after the production of milk, and they could, thus, be regulated appropriately in order to reduce bacterial load and improve the quality of milk delivered to dairy plants.

## Figures and Tables

**Table 1:** Frequency of staphylococcal isolates (presented by bacterial species) recovered from the bulk-tank milk of 444 small ruminant farms in Greece, in accord with the type of animals in the farm (sheep or goats) and the milking mode applied (machine- or hand-milking).

Staphylococcal Isolates	Frequency of Isolates (*n*) (Proportion of Isolation ^1^)			
	Animals in Farms		Milking Mode in Farms	
	Sheep ^1^	Goats	Machine-Milking	Hand-Milking
*S. aureus*	54 (23.3%)	21 (26.3%)	51 (22.5%)	24 (28.2%)
*S. auricularis*	3 (1.3%)	1 (1.3%)	2 (0.9%)	2 (2.4%)
*S. capitis*	6 (2.6%)	6 (7.5%)	7 (3.1%)	5 (5.9%)
*S. carnosus*	2 (0.9%)	0 (0.0%)	2 (0.9%)	0 (0.0%)
*S. chromogenes*	13 (5.6%)	1 (1.3%)	9 (4.0%)	5 (0.8%)
*S. cohnii* subsp. *cohnii*	4 (1.7%)	1 (1.3%)	5 (2.2%)	0 (0.0%)
*S. cohnii* subsp. *urealyticum*	3 (1.3%)	2 (2.5%)	4 (1.8%)	1 (1.2%)
*S. epidermidis*	4 (1.7%)	1 (1.3%)	5 (2.2%)	0 (0.0%)
*S. equorum*	23 (9.9%)	11 (13.8%)	23 (10.1%)	11 (12.9%)
*S. haemolyticus*	22 (9.5%)	4 (5.0%)	22 (9.7%)	4 (4.7%)
*S. hominis*	2 (0.9%)	1 (1.3%)	1 (0.4%)	2 (2.4%)
*S. intermedius*	6 (2.6%)	1 (1.3%)	3 (1.3%)	4 (4.7%)
*S. kloosii*	7 (3.0%)	3 (3.8%)	8 (3.5%)	2 (2.4%)
*S. lentus*	12 (5.2%)	5 (6.3%)	14 (6.2%)	3 (3.5%)
*S. lugdunensis*	11 (4.7%)	2 (2.5%)	11 (4.8%)	2 (2.4%)
*S. pasteuri*	2 (0.9%)	0 (0.0%)	2 (0.9%)	0 (0.0%)
*S. pettenkoferi*	0 (0.0%) ^2^	3 (3.8%) ^2^	2 (0.9%)	1 (1.2%)
*S. saprophyticus*	4 (1.7%)	0 (0.0%)	3 (1.3%)	1 (1.2%)
*S. sciuri*	3 (1.3%)	0 (0.0%)	3 (1.3%)	0 (0.0%)
*S. simulans*	35 (15.1%)	9 (11.3%)	33 (14.5%)	11 (12.9%)
*S. vitulinus*	3 (1.3%)	4 (5.0%)	4 (1.8%)	3 (3.5%)
*S. warneri*	9 (3.9%)	2 (2.5%)	9 (4.0%)	2 (2.4%)
*S. xylosus*	4 (1.7%)	2 (2.5%)	4 (1.8%)	2 (2.4%)
Total	232	80	227	85

^1^ In brackets, proportion of isolation of each species among all staphylococcal species recovered from respective farms; ^2^ *p* = 0.004.

**Table 2:** Frequency of staphylococcal species recovered from the bulk-tank milk of 444 small ruminant farms in Greece and frequency and proportion of isolates that were biofilm forming.

Staphylococcal Isolates	No. of Isolates	No. of Biofilm-Forming Isolates	Proportion of Biofilm-Forming Isolates among Species	*p*-Value
*S. aureus*	75	58	77.3%	0.22
*S. auricularis*	4	4	100.0%	0.21
*S. capitis*	12	10	83.3%	0.37
*S. carnosus*	2	2	100.0%	0.37
*S. chromogenes*	14	9	64.3%	0.52
*S. cohnii* subsp. *cohnii*	5	2	40.0%	0.11
*S. cohnii* subsp. *urealyticum*	5	4	80.0%	0.68
*S. epidermidis*	5	4	80.0%	0.68
*S. equorum*	34	24	70.6%	0.87
*S. haemolyticus*	26	12	46.2%	0.002
*S. hominis*	3	2	66.7%	0.84
*S. intermedius*	7	4	57.1%	0.38
*S. kloosii*	10	9	90.0%	0.19
*S. lentus*	17	10	58.8%	0.22
*S. lugdunensis*	13	5	38.5%	0.006
*S. pasteuri*	2	2	100.0%	0.37
*S. pettenkoferi*	3	3	100.0%	0.28
*S. saprophyticus*	4	4	100.0%	0.21
*S. sciuri*	3	3	100.0%	0.28
*S. simulans*	44	32	72.7%	0.88
*S. vitulinus*	7	6	85.7%	0.41
*S. warneri*	11	10	90.9%	0.15
*S. xylosus*	6	5	83.3%	0.53
Total	312	224	71.8%	

**Table 3:** Multivariable analysis for variables significantly associated with the isolation of biofilm-forming staphylococci from the bulk-tank milk in small ruminant farms in Greece, with separate analysis for farms applying machine-milking or hand-milking.

Variable	Odds Ratios ^1^ (95% Confidence Intervals)	*p* Value
Farms applying machine-milking		
Temperature of the water used to clean the milking parlour		0.024
<50 °C (88.9% ^2^)	12.507 (2.791–56.046)	0.001
50–69 °C (47.9%)	1.438 (0.873–2.369)	0.15
70–89 °C (39.0%)	reference	-
≥90 °C (56.0%)	1.990 (0.856–4.628)	0.11
Farms applying hand-milking		
Frequency of milk collection from the tank		0.08
Daily (38.1%)	reference	-
At least every two days (48.5%)	1.526 (0.992–2.346) ^2^	0.05

^1^ Odds ratios calculated against the lowest prevalence associations of the variable. ^2^ Proportion of farms among those in which the studied variable prevailed, in which biofilm-forming staphylococci were isolated from bulk-tank milk.

## Data Availability

Most data presented in this study are in the text or in the Supplementary Materials. The remaining data are available on request from the corresponding author. The data are not publicly available as they form part of the PhD thesis of the first author, which has not yet been examined, approved and uploaded in the official depository of PhD theses from Greek Universities.

## Supplementary Materials

The following supporting information can be downloaded at: https://www.mdpi.com/article/10.3390/foods12152836/s1, Table S1: Details of multivariable models employed for the evaluation for potential associations of the recovery of biofilm-forming staphylococci from the bulk-tank of milk in 444 small ruminant farms in Greece; Table S2: Cumulative information collected from 444 small ruminant farms in a countrywide investigation in Greece, classified according to animal species in the farms; Table S3: Results of univariable analysis for associations of recovery of biofilm-forming staphylococcal isolates from bulk-tank milk with the season of the year when sampling was performed in 444 small ruminant farms in Greece; Table S4: Isolation of biofilm-forming staphylococci from small ruminant farms during a countrywide investigation in Greece, in accord with the temperature of the water used to clean the milking parlour; Table S5: Results of univariable analysis for associations of recovery of biofilm-forming staphylococcal isolates from bulk-tank milk with management practices applied in 444 small ruminant farms in Greece; Table S6: Parameters of the bulk-tank milk of 444 small ruminant farms in Greece in accord with the isolation of biofilm-forming staphylococci from the milk.

## Appendix A

**Table A1:** Details of management-related variables (*n* = 11) used in the evaluations for potential associations with recovery of biofilm-forming staphylococcal isolates from bulk-tank milk in 444 small ruminant farms during a countrywide investigation in Greece.

Management system applied in the farm (description (intensive, semi-intensive, semi-extensive, extensive) according to the classification of the European Food Safety Authority [35])
No. of female animals in the farm (no.)
Type of milking (hand-milking/machine-milking)
Annual frequency of changing teatcups (no. of occasions)
Temperature of cleaning water of the milk parlour (°C)
Frequency of milk collection from the tank (days)
Daily number of milking sessions (no.)
Use of teat disinfection after milking (yes/no)
Administration of ‘dry-ewe’ treatment at the end of the lactation period (yes/no)
Annual frequency of systemic disinfections in the farm
Month into the lactation period at sampling
