# Supplementary material for: Isolation of Biofilm-Forming Staphylococci from the Bulk-Tank Milk of Small Ruminant Farms in Greece
Source: Foods. 2023 Jul 26;12(15):2836. doi: 10.3390/foods12152836 (PMC10417191; doi:10.3390/foods12152836)
Supplement: Supplementary file 1 [file foods-12-02836-s001.zip › foods-2496553-supplementary.pdf]

Isolation of Biofilm-Forming Staphylococci from the Bulk-Tank Milk of Small Ruminant Farms in Greece

Daphne T. Lianou, Charalambia K. Michael, Nikolaos Solomakos, Natalia G. C. Vasileiou, Efthymia Petinaki, Vasia S. Mavrogianni, Athina Tzora, Chrysoula Voidarou, and George C. Fthenakis

Table S1. Details of multivariable models employed for the evaluation for potential associations of the recovery of biofilm-forming staphylococci from the bulk-tank of milk in 444 small ruminant farms in Greece.

Outcome	Variables (*n*)	
	**offered to the multi-variable models**	**required in the final models**
Isolation of biofilm-forming staphylococci - only farms applying machine-milking	4 [1]	(a) Temperature of cleaning water of the milking parlour, (b) Frequency of milk collection from the tank
Isolation of biofilm-forming staphylococci - farms applying hand-milking	3 [2]	(a) Season of the year when sampling was carried out, (b) Frequency of milk collection from the tank, (c) Use of teat disinfection after milking

[1] Season of the year when sampling was performed, Temperature of cleaning water of the milking parlour, Frequency of milk collection from the tank, Use of teat disinfection after milking; [2] Season of the year when sampling was performed, Frequency of milk collection from the tank, Use of teat disinfection after milking.

Table S2. Cumulative information collected from 444 small ruminant farms in a countrywide investigation in Greece, classified according to animal species in the farms.

	Sheep flocks (*n* = 325)	Goat herds (*n* = 199)
Management system applied in the farm	Intensive: 44, semi-intensive: 140, semi-extensive: 116, extensive: 25	Intensive: 9, semi-intensive: 29, semi-extensive: 61, extensive: 20
No. of female animals in the farm	$\bar{X}$: 325 ± 13 ewes	$\bar{X}$: 237 ± 20 does
Type of milking	Machine-milking: 255, hand-milking no: 70	Machine-milking: 66, hand-milking: 53
Annual frequency of changing teatcups	Once: 130, less than once: 125	Once: 29, less than once: 37
Temperature of cleaning water of the milking parlour	$\bar{X}$: 70 ± 0.5 ^{o}C	$\bar{X}$: 70 ± 1.0 ^{o}C
Frequency of milk collection from the tank	Daily: 82, at least every two days: 243	Daily: 36, at least every two days: 83
Daily number of milking sessions	2 (1 – 3)	2 (1 – 3)
Use of teat disinfection after milking	Yes: 52, no: 273	Yes: 12, no: 107
Administration of 'dry-ewe' treatment at the end of the lactation period	Yes: 53, no: 272	Yes: 15, no: 104
Annual frequency of systemic disinfections in the farm	$\bar{X}$: 3.6 ± 0.2 occasions	$\bar{X}$: 4.3 ± 0.5 occasions
Month into the lactation period at sampling	0-1st: 23, 2nd-5th: 138, 6th-9th: 147, over 9th: 17	0-1st: 8, 2nd-5th: 60, 6th-9th: 43, over 9th: 8

Table S3. Results of univariable analysis for associations of recovery of biofilm-forming staphylococcal isolates from bulk-tank milk with the season of the year when sampling was performed in 444 small ruminant farms in Greece.

**Recovery of biofilm-forming staphylococcal isolates (*n* = 203)**				**No recovery of biofilm-forming staphylococcal isolates (*n* = 241)**				***p***
**Season of the year when sampling was performed**								
Autumn	Winter	Spring	Summer	Autumn	Winter	Spring	Summer	
18	50	82	53	23	61	69	88	0.040

Table S4. Isolation of biofilm-forming staphylococci from small ruminant farms during a countrywide investigation in Greece, in accord with the temperature of the water used to clean the milking parlour.

Temperature ranges	Proportion of farms from which biofilm-forming staphylococci were isolated	*p*-value	Median (interquartile range) number of biofilm-forming staphylococci isolated per farm	*p*-value
< 49 ^{o}C	88.9%	0.0002	1 (0)	< 0.0001
50 ^{o}C – 69 ^{o}C	47.9%	0.62	0 (1)	0.70
70 ^{o}C – 89 ^{o}C	39.0%	0.005	0 (1)	0.005
≥ 90 ^{o}C	56.0%	0.29	1 (1)	0.34

Table S5. Results of univariable analysis for associations of recovery of biofilm-forming staphylococcal isolates from bulk-tank milk with management practices applied in 444 small ruminant farms in Greece.

**Recovery of biofilm-forming staphylococcal isolates (*n* = 203)**				**No recovery of biofilm-forming staphylococcal isolates (*n* = 241)**				***p***
**Management system applied in the farm**								
Intensive	Semi-intensive	Semi-extensive	Extensive	Intensive	Semi-intensive	Semi-extensive	Extensive	
24	81	83	15	29	88	94	30	0.36
**No. of female animals in the farm**								
≤ 165	166 - 330	331 – 500	> 500	≤ 165	166 - 330	331 – 500	> 500	
66	78	30	29	79	79	49	35	0.41
**Type of milking**								
Machine-milking		Hand-milking		Machine-milking		Hand-milking		
147		56		174		67		0.96
**Annual frequency of changing teatcups**								
Up to annually		Less often than annually		Up to annually		Less often than annually		
70		77		89		85		0.53
**Temperature of cleaning water of the milking parlour (°C)**								
< 49	50 – 69	70 – 89	≥ 90	< 49	50 – 69	70 – 89	≥ 90	
16	46	71	14	2	50	111	11	0.0004
**Frequency of milk collection from the tank (days)**								
Daily		At least every two days		Daily		At least every two days		
45		158		73		168		0.05
**Daily number of milking sessions**								
One	Two	Three		One	Two	Three		
2	167	34		3	205	33		0.65
**Use of teat disinfection after milking**								
Yes		No		Yes		No		
34		167		30		213		0.17
**Administration of 'dry-ewe' treatment at the end of the lactation period**								
Yes		No		Yes		No		
35		168		33		208		0.30
**Annual frequency of systemic disinfections in the farm**								
Median: 2 (interquartile range: 2)				Median: 2 (interquartile range: 2)				0.38

Month into the lactation period at sampling								
0-1st	2nd-5th	6th-9th	Over 9th	0-1st	2nd-5th	6th-9th	Over 9th	
16	89	89	9	15	109	101	16	0.68

Table S6. Parametres of the bulk-tank milk of 444 small ruminant farms in Greece in accord with the isolation of biofilm-forming staphylococci from the milk.

Parameters	Isolation of biofilm-forming staphylococci	Isolation of non biofilm- forming staphylococci	No isolation of staphylococci	ls means (sem)	*p*-value
Sheep flocks (n = 325)					
Somatic cell counts [1]	0.575×10^6 (0.518×10^6 - 0.638×10^6) cells mL^{-1}	0.503×10^6 (0.430×10^6 - 0.587×10^6) cells mL^{-1}	0.385×10^6 (0.333×10^6 - 0.442×10^6) cells mL^{-1}	0.479×10^6 (0.013×10^6) cells mL^{-1}	< 0.0001
Total bacterial counts [1]	499×10^3 (372×10^3 - 676×10^3) cfu mL^{-1}	378×10^3 (245×10^3 - 575×10^3) cfu mL^{-1}	308×10^3 (240×10^3 - 398×10^3) cfu mL^{-1}	387×10^3 (1×10^3) cfu mL^{-1}	0.046
Fat content [2]	6.08% ± 0.07%	6.17% ± 0.11%	6.26% ± 0.08%	6.17% (0.03%)	0.22
Protein content [2]	4.42% ± 0.02%	4.46% ± 0.03%	4.42% ± 0.02%	4.43% (0.01%)	0.59
Lactose content [2]	4.19% ± 0.02%	4.24% ± 0.03%	4.21% ± 0.02%	4.22% (0.02%)	0.41
Goat herds (n = 119)					
Somatic cell counts [1]	0.879×10^6 (0.764×10^6 - 1.009×10^6) cells mL^{-1}	0.871×10^6 (0.689×10^6 - 1.104×10^6) cells mL^{-1}	0.777×10^6 (0.647×10^6 - 0.935×10^6) cells mL^{-1}	0.842×10^6 (0.013×10^6) cells mL^{-1}	0.53
Total bacterial counts [1]	633×10^3 (417×10^3 - 955×10^3) cfu mL^{-1}	723×10^3 (372×10^3 - 1413×10^3) cfu mL^{-1}	507×10^3 (339×10^3 - 759×10^3) cfu mL^{-1}	608×10^3 (1×10^3) cfu mL^{-1}	0.62
Fat content [2]	4.78% ± 0.15%	4.38% ± 0.26%	4.93% ± 0.20%	4.73% (0.09%)	0.25
Protein content [2]	3.20% ± 0.03%	3.22% ± 0.05%	3.27% ± 0.06%	3.25% (0.02%)	0.56
Lactose content [2]	4.77% ± 0.04%	4.82% ± 0.05%	4.67% ± 0.06%	4.74% (0.01%)	0.14

[1] mean (95% confidence intervals); [2] mean ± standard error of the mean.
